# Intestinal Transcriptome Analysis Reveals Enrichment of Genes Associated with Immune and Lipid Mechanisms, Favoring Soybean Meal Tolerance in High-Growth Zebrafish (*Danio Rerio*)

**DOI:** 10.3390/genes12050700

**Published:** 2021-05-08

**Authors:** Luis Valenzuela, Sebastian Pacheco, Gonzalo Rincón, Leonardo Pavez, Natalia Lam, Adrián J. Hernández, Patricio Dantagnan, Felipe González, Felipe Jilberto, M. Cristina Ravanal, Cecilia Ramos, Héctor Garcia, Cristian Araneda, Pilar E. Ulloa

**Affiliations:** 1 Omics Lab, Villavicencio 378, Oficina 32, Santiago 8320164, Chile; luis.valenz.v@gmail.com; 2 Programa de Doctorado en Inmunología y Microbiología, Universidad San Sebastian, Lota 2465, Santiago 7510157, Chile; spachecoa@correo.uss.cl; 3 Zoetis, VMRD Genetics R&D, 333 Portage Street, Kalamazoo, MI 49007, USA; grincon@ucdavis.edu; 4 Núcleo de Investigaciones Aplicadas en Ciencias Veterinarias y Agronómicas, Universidad de Las Américas, Avenida Manuel Montt 948, Santiago 7500975, Chile; lpavez@udla.cl (L.P.); gonzalez.iturriaga.felipe@gmail.com (F.G.); cramosb@udla.cl (C.R.); 5 Departamento de Producción Animal, Facultad de Ciencias Agronómicas, Universidad de Chile, Santa Rosa 11315, Santiago 8820808, Chile; nlam@uchile.cl (N.L.); fjilberto@ug.uchile.cl (F.J.); craraned@uchile.cl (C.A.); 6 Laboratorio de Nutrición y Fisiología de Peces, Núcleo de Investigación en Producción Alimentaria, Departamento de Ciencias Agropecuarias y Acuícolas, Facultad de Recursos Naturales, Universidad Católica de Temuco, Temuco 4780000, Chile; ajhernandez@uct.cl (A.J.H.); dantagna@uct.cl (P.D.); 7 Instituto de Ciencia y Tecnología de los Alimentos (ICYTAL), Facultad de Ciencias Agrarias y Alimentarias, Universidad Austral de Chile, Avda. Julio Sarrazín s/n, Isla Teja, Valdivia 5090000, Chile; cristina.ravanal@gmail.com; 8 Laboratorios Diagnofruit Ltd.a., Depto. Fitopatología Molecular, Santiago 7770273, Chile; hgarcia@diagnofruit.cl

**Keywords:** zebrafish, transcriptome, RNA-seq, soybean meal tolerance, high-growth fish, sustainable aquaculture

## Abstract

The molecular mechanisms underlying fish tolerance to soybean meal (SBM) remain unclear. Identifying these mechanisms would be beneficial, as this trait favors growth. Two fish replicates from 19 experimental families were fed fishmeal-(100FM) or SBM-based diets supplemented with saponin (50SBM + 2SPN) from juvenile to adult stages. Individuals were selected from families with a genotype-by-environment interaction higher (HG-50SBM + 2SPN, 170 ± 18 mg) or lower (LG-50SBM + 2SPN, 76 ± 10 mg) weight gain on 50SBM + 2SPN for intestinal transcriptomic analysis. A histological evaluation confirmed middle intestinal inflammation in the LG- vs. HG-50SBM + 2SPN group. Enrichment analysis of 665 differentially expressed genes (DEGs) identified pathways associated with immunity and lipid metabolism. Genes linked to intestinal immunity were downregulated in HG fish (*mpx*, *cxcr3.2*, *cftr*, *irg1l*, *itln2*, *sgk1*, *nup61l, il22*), likely dampening inflammatory responses. Conversely, genes involved in retinol signaling were upregulated (*rbp4*, *stra6*, *nr2f5*), potentially favoring growth by suppressing insulin responses. Genes associated with lipid metabolism were upregulated, including key components of the SREBP (*mbtps1*, *elov5l*, *elov6l*) and cholesterol catabolism (*cyp46a1*), as well as the downregulation of *cyp7a1*. These results strongly suggest that transcriptomic changes in lipid metabolism mediate SBM tolerance. Genotypic variations in DEGs may become biomarkers for improving early selection of fish tolerant to SMB or others plant-based diets.

## 1. Introduction

The aquaculture industry has a growing demand for alternative plant-based ingredients to complement the nutritional value of aquafeeds. Reliance on marine protein and lipid sources for commercial aquafeeds has significantly declined, largely due to record fishmeal (FM) (US $$ 1919/ton) and fish oil ($2300/ton) costs in recent years [1]. Given the projected growth in farmed fish production (~18% by 2030) [1], aquafeed formulations may come to depend heavily on diverse plant proteins as well as new animal protein sources [1]. To ensure its viability, the industry must diversify and optimize plant ingredients and inclusion levels in feeds for carnivorous species (e.g., salmonids) without affecting the productive or physiological performance of the fish. Unfortunately, numerous studies have reported that diets containing soybean meal (SBM) and/or other plant proteins typically induce intestinal inflammation and decrease growth in carnivorous species, such as Atlantic salmon (*Salmo salar*) [2] and rainbow trout (*Oncorhynchus mykiss*) [3], as well as in omnivores, such as the common carp (*Cyprinus carpio*) [4] and zebrafish (*Danio rerio*) [5], hindering growth [6,7]. This deleterious intestinal response is attributable partly to antinutritional factors (ANFs) present in soybeans, but primarily to soy saponin as allergen to cause this pathology [5,7,8,9]. Seabream (*Sparus aurata*) fed a diet containing ≥65% plant ingredients showed 20% less growth than fish on a FM-based diet [10]. Similar results have been found in tilapia (*Oreochromis niloticus*) ([11], rainbow trout) [12,13], Atlantic salmon [14], and zebrafish [15], with 40%, 25%, 13%, and 21% less growth, respectively. 

Promisingly, however, different results have been described even among fish of the same species fed the same percentage SBM. For example, while Krogdahl et al. [16] reported that a diet containing 20% SBM reduced Atlantic salmon growth by 15%, Overland et al. [17] found that a similar feed, also containing 20% SBM, had no effect on growth in Atlantic salmon, even after 84 days on the diet. In another study, rainbow trout fed a 50% SBM diet did not develop intestinal inflammation and showed no significant difference in weight gain vs. the control group on a FM diet (182 g vs. 178 g) [18]. Authors have attributed these divergent results primarily to differences in environmental or other conditions (such as soybean strain or cultivar, feed formulation, amino acid profile, pellet digestibility, experimental design, or fish size). The potential role of genetic background in these outcomes, however, has been scarcely studied. 

Growth is an important trait in aquaculture production, due to its intrinsic link with productivity and profitability in industry. Inheritance of growth rate is governed by many loci, and the presence of major genes (quantitative trait loci, QTL) is affected by environmental factors [19]. Growth heritability is estimated at ~41% for farmed fish and zebrafish, supporting a conservative influence of genetics on this phenotypic trait [20,21]. Selective breeding programs typically aim to optimize growth by elucidating the genetic underpinnings of this trait of interest.

Whole transcriptome analysis, via technologies, such as RNA-sequencing (RNA-seq), can be used to evaluate gene expression profile, identifying relevant genes and single-nucleotide polymorphisms (SNPs) in the gene sequence [22]. In the present case, RNA-seq data can be used to identify biological processes and metabolic pathways involved in growth.

At the transcriptional level, microarray analysis has been used to make great strides towards understanding the effects of plant proteins on the intestinal or hepatic health of Atlantic salmon [9,23,24], rainbow trout [25], and juvenile yellow perch (*Perca flavescens*) [26]. Most such studies have examined transcriptional changes after dietary FM is replaced with plant-based proteins, analyzing the intestinal pathology and molecular mechanisms implicated during early onset and progression of symptoms [9,23,24]. In general, inflammation is characterized by upregulation of immune-related genes, including cytokines, GTPase IMAP family members, NF-kB-related genes, TNF α related genes, and regulators of T and B cell function. A hallmark of metabolic changes is dramatic downregulation of lipid, bile, and steroid metabolism, reflecting digestive and metabolic dysfunction [9,23,24]. Marked downregulation of xenobiotic metabolism has been also observed, possibly increasing the vulnerability of the intestinal tissue and activating multiple defense processes that participate in mucosal healing and restoration of normal tissue function [9]. 

Other studies have compared regimens, such as SBM versus mixed-plant diets containing corn gluten, sunflower, rapeseed, or horsebean and soy protein concentrate or fava bean concentrate. Studies have evaluated the respective impact of such diets on intestinal inflammation, evaluating for common or distinct gene expression patterns and links between such patterns and fish performance [7,9]. Results have shown that different plant protein materials generate differential gut- gene expression patterns (genes, pathways, and GO terms). Diets that include corn gluten, sunflower, rapeseed, or horsebean [9], as well as mixed diets containing soy protein or fava bean concentrate [7], produce less marked transcriptomic alterations and result in more favorable body composition than diets containing a combination of saponins and pea protein [9]. SBM is known to cause major transcriptome changes associated with enteritis and, therefore, decreased body weight [7]. Importantly, these studies provide evidence of a link between the magnitude of intestinal transcriptomic changes and the differences in growth rate in response to plant diets. Consequently, a key step towards ensuring efficient fish growth is identifying genes that confer intestinal tolerance to alternative protein sources, such as SBM, that are to be incorporated into aquafeed formulations. A familial design for this type of study can facilitate selection of the fish with the most marked heritable variations. 

Zebrafish is an excellent model organism for studying nutrition [27,28], gut immunity [29,30], and nutritional genomics, with applications in commercial aquaculture [31]. The fully sequenced zebrafish genome allows for RNA-seq assays that can be useful to identify molecular mechanisms underlying responses to various nutrients [31]. Example is the identification of SNPs in candidate genes in muscle of zebrafish fed SBM based diet associated to growth [32]. Zebrafish have been found to mirror the biological responses observed in commercial fish fed an SBM-based diet, including intestinal inflammation [5,33] and decreased growth [15]. Moreover, studies have shown that incorporating protective intestinal additives in a SBM diet decreases intestinal inflammation [34,35] like those of commercially important fish. As omnivores, zebrafish can feed on plant and on animal proteins sources, suggesting on similar digestive mechanism as do some omnivore or carnivore commercial fish [31]. 

Unlike previous studies, we analyzed two replicates from 19 experimental families fed a fishmeal-(100FM) or SBM-based diet supplemented with saponin (50SBM + 2SPN) from the juvenile to adult stages. The fish were selected from families that showed a genotype-by-environment interaction in response to the 50SBM + 2SNP vs. 100FM diets. This interaction suggests that families with the greatest weight gain on the 50SBM + 2SPN may harbor genes that confer greater tolerance to SBM, thus favoring growth. 

Individuals with the higher (HG-50SBM + 2SPN, 170 ± 18 mg) and lower (LG-50SBM + 2SPN, 76 ± 10 mg) weight gain on the SBM-based diets, respectively, were selected for further analysis. Tolerance refers here to the ability of an organism to digest certain substances, such as SBM, without suffering effects on wellness and growth. Low growth, in contrast, presumably indicates nutritional stress.

Given that these digestive mechanisms are conserved among fish species, the experimental results enrich our understanding of molecular mechanisms that favor growth on a plant-based diet. Differentially expressed genes could be used as biomarkers to identify SNPs for evaluating intestinal tolerance in commercial fish fed with SBM-based diets. Thus, contribute to the long-term stability of the aquaculture industry. 

## 2. Materials and Methods

### 2.1. Generation of Zebrafish Population

A population of 19 families was generated from three wild-type strains from different laboratories (♀ TAB-5 from the United States and ♂ DB-3 or AB from Chile) to optimize genetic variability. The biological material was provided by the Danio Biotech Company (Santiago, Chile), Zebrafish for Innovation and Research Laboratory (ZIRLab), Faculty of Sciences, University of Chile. Families were grown simultaneously, from the egg production to larval period (6–40 days post fertilization, dpf), prior to the experimental assay. Each family used in this study had an egg fertilization rate of at least 80% at 48 h post-fertilization and 80% larval survival at 5 dpf (at least 60 larvae). At the end of the larval period (40 dpf), all families had a survival rate of 85% with at least 52 surviving larvae before the experimental assay was initiated. All animal-handling procedures were conducted in compliance with the zebrafish welfare terms of protocol No. 1837-FCS-UCH and were approved by the Institutional Committee for the Care and Use of Animals at the University of Chile (CICUA, BIO 19003).

### 2.2. Fish Rearing and Experimental Diet

During the juvenile stage (40 dpf), each family was split to generate two replicates (23 fish per family), thus creating two fish populations with similar genetic backgrounds (19 families × 23 fish, total *n* = 437). Twenty-three fish from each family were randomly distributed into thirty-eight 14 L tanks with recirculating water and kept at a density of 600 cm^3^ fish^−1^. The fish were acclimatized to the new system for five days and fed a commercial diet of 300-μm Gemma Micro^®^ (Skretting, Osorno, Chile) pellets. 

The first population was fed the experimental diet containing 50% soybean meal supplemented with 2 g/kg saponin (50SBM + 2SPN) (certified 95% pure; obtained from Organic Technologies, Coshocton, Ohio, USA) from the juvenile (45 dpf) to adult (98 dpf) stages. Studies have shown that the saponin present in SBM is the causal agent of enteritis in fish [5,8,9]. Prior analyses have found saponin levels in the range of 5−7 g/kg in SBM [36]. A SBM content of 50% translates to saponin levels of ca. ~3 g/kg; in a previous study, these levels did not produce statistically significant inflammatory effect in zebrafish larvae, according to histology, during early feeding [5]. Therefore, the SBM diet used in the present study was supplemented with saponin to achieve levels of ~4.8 g/kg, which was sufficient to induce inflammatory changes in juvenile Atlantic salmon over 10 weeks of feeding [9]. The second population (19 families × 23, total *n* = 437) was fed a control diet with fishmeal as the primary protein source (100FM) as a negative control for inflammation. All fish were fed ad *libitum* three times daily for 53 days. Table 1 presents the ingredients and nutrient composition of the control and experimental diets. Diets were supplemented with a standard vitamin and mineral premix and formulated to be isoenergetic (20.0 for 100FM vs. 20.3 for 50SBM + 2SNP), isonitrogenous (46.4 for 100FM vs. 43.5 for 50SBM + 2SPN), and isolipidic (7.8 for 100FM vs. 8.4 for 50SBM + 2SNP). Additionally, the diets fulfilled the essential amino acid requirements of cyprinid fish [37]; 50SBM + 2SPN vs. 100FM: 2.98 vs. 2.65% arginine, 1.27 vs. 1.35% histidine, 2.17 vs. 2.09% isoleucine, 3.54 vs. 3.52% leucine, 3.25 vs. 3.62% lysine, 2.19 vs. 1.96% phenylalanine, 1.25 vs. 0.92% methionine, 1.84 vs. 1.90% threonine, 1.54 vs. 1.44% tyrosine, and 2.40 vs. 2.45% valine. Both diets were manufactured at the Animal Feed Pilot Plant (Department of Agriculture and Aquaculture Sciences, Natural Resources Faculty, Catholic University of Temuco) using a twin-screw extruder (CLEXTRAL BC-21, Clextral, Firminy, France) that produces 2 mm diameter pellets, then coated with fish oil using a laboratory vacuum coater (Dinnissen Model VC10, Sevenum, Netherlands). The pellets were subsequently crumbled and screened to the appropriate particle size (350–500 μm) and stored at −20 °C until use in the experimental feeding trials.

During all growth periods, the fish were reared in water with optimal physical and chemical parameters (27–28 °C, pH 6.8–7.3, conductivity 350–500 μs/cm, nitrites 0 mg/L, nitrates ≤50 mg/L) and a 14 h light:10 h dark photoperiod [38]. All fish were reared in the zebrafish facilities at the Aquaculture Laboratory of Genetics and Biotechnology, Animal Production Department, Faculty of Agronomy, University of Chile.

### 2.3. Growth Measurement and Intestinal Samples

At 40 dpf, a sample of 20 fish per family was used to record initial weight and length (total *n* = 380). Weight gain (WG) and length gain (DL) and Specific Growth Rate (SGR) were recorded at 98 dpf in all fish from each family in each population. WG and DL were calculated as the difference between the measurements at the two stages (WG (mg) = weight at 98 dpf -initial weight at 40 dpf; DL (mm) = length at 98 dpf—initial length at 40 dpf. SGR was calculated using the following equation: 100%*(ln weight at 98dpf—ln initial weight at 40 dpf)/t, where t is the number of experimental days. The fish were fasted for 24 h prior to each weighing event and weighed on a scale calibrated to the nearest 0.001 g (Acculab VI-3 mg). Length (mm) was measured from mouth to caudal peduncle using digital photography and ImageJ analysis software (TPSdig2 v2. 12). To avoid potential confounding effects of gender in the transcriptome analysis, only males were considered. Gender was determined by examination of external secondary sexual characteristics, including anal fin and ventral body surface coloration (yellow in males) and body shape (rounded belly in females) [39]. The fish were sacrificed to obtain intestinal samples. Individual intestines were placed in a labeled 1.5 mL polypropylene tube containing RNA-later solution and stored at −80 °C until RNA extraction. All animal-handling procedures were conducted according to Zebrafish Care and Management Protocol N°51, approved by the Institutional Committee for the Care and Use of Animals at the University of Chile (CICUA, 18190 AGR-UCH). At 40 dpf, the data were analyzed by one-way analysis of variance (ANOVA) with the Waller–Duncan multiple range test (*p*-value < 0.05). The data at 98 dpf were analyzed by two-way analysis of variance (ANOVA) with the Student–Newman–Keuls multiple comparison test to evaluate fixed effects (diet and family) using the SPSS V17.0 GML procedure.

### 2.4. Selection of Fish for RNA Sequencing

**Figure 1:**
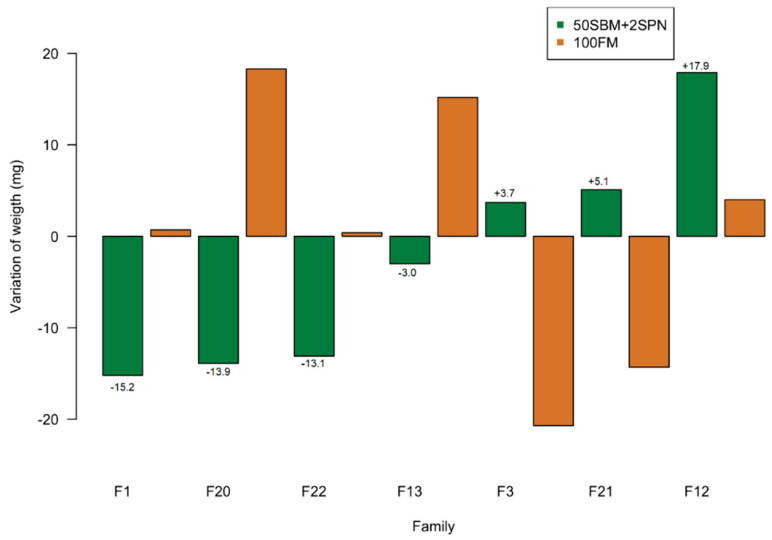
Variation in weight gain of families that showed a genotype-by-environment interaction in response to 50SBM + 2SNP vs. 100FM diets. Families with the greatest (F3, F21, and F12) and least weight gain (F1, F20, F22, and F13) in terms of deviation from the 50SBM + 2SPN population mean (120 ± 33 mg).

Individuals were selected for RNA-seq according to growth phenotype (WG) at the end of the experiment (98 dpf). The fish were selected from families that showed a genotype-by-environment interaction in response to the 50SBM + 2SNP vs. 100FM diets (Figure 1). Families with the greatest WG on the 50SBM + 2SPN vs. 100FM diets may harbor genes that confer greater tolerance to the 50SBM + 2SPN diet, thus favoring growth. The differential WG for the families with the greatest gains (F3, F21, and F12) in terms of deviation from the 50SBM + 2SPN population mean (120 ± 33 mg) was +3.7; +5.1; and +17.9 mg, respectively. The differential WG for the families with the smallest gain (F1, F20, F22, and F13) was −15.2; −13.9; −13.1; and −3.0 (Figure 1). From each group of families, five fish with greater WG (average = 170 ± 18 mg) and six fish with smaller gains (average = 76 ± 10 mg) were selected for RNA-seq analysis (Table 2).

Additionally, relatedness between parents in each family was estimated using SSR genotyping (zf644, z732, z1197, z1637, z4009, z4830, z4951, z5058, and z5294) [40]. In the HG families used for RNA-seq, the three pairs of parents were found to be unrelated (rXY = 0.000); in the LG families, the four pairs of parents were found to be second cousins (rXY = 0.037 to 0.057), contributing to genetic variability among phenotypes.

### 2.5. RNA Extraction and Library Preparation

Total RNA was extracted from the intestines of individual fish. The tissue was homogenized in TRIzol*^®^* (Invitrogen, Carlsbad, CA, USA), following the manufacturer’s protocol. RNA concentration, quality, and integrity were assessed according to the 260/280 ratio (>1.7) and RQN (>7.5) by capillary electrophoresis with a Fragment Analyzer Automated CE System. All samples were previously treated with Turbo DNAse I (Invitrogen) to remove any genomic DNA contamination.

Eleven RNA-seq libraries (five of HG-50SBM + 2SBM and six of HG-50SBM + 2SBM samples) were constructed using TruSeq Stranded mRNA LT Sample Prep Kit following the TruSeq Stranded mRNA Sample Preparation Guide, Part #15031047 Rev. E. Briefly, each sequencing library was prepared by random fragmentation of the cDNA sample, followed by 5′ and 3′ adapter ligation. Alternatively, “tagmentation” combines the fragmentation and ligation reactions into a single step that greatly increases the efficiency of the library preparation process. Adapter-ligated fragments were then PCR-amplified and gel purified. The fragments were sequenced with a read length of 150 base pairs using Illumina sequencing technology.

### 2.6. Sequencing Data Processing and Differential Gene Expression

Each one of the libraries was processed as follows: using QIAGEN CLC Genomics Workbench 20.0.3, adapters from the raw FASTQ files were removed, and the raw reads with a Q (Phred) quality index greater than 30 (Q > 30) were selected. The Phred quality score numerically expresses the accuracy of each sequenced nucleotide; if Phred assigns a quality score of 30 to a base, the chances of a base-call error are one in 1000. The resulting high-quality reads were mapped against the reference genome assembly for zebrafish GRCz11 (GenBank assembly accession GCF_000002035.6). Transcripts Per Million were calculated using raw counts from the aligned reads (TPM = 106*RPKM (reads per kilobase of transcript per million reads mapped)/sum (RPKM)) [41,42]. The similarity of transcriptomic profiles among all samples was evaluated from the TPM data, via Principal Component Analysis (PCA) and correlation matrices, using the R ADE4 package. Then, within each phenotype, five individuals and their respective TPM values were used to perform a differential expression analysis using the Wald test in CLC Genomics Workbench. Genes with *p*-values ≤0.01 and fold change values ≤−1.5 and ≥1.5 were considered DEGs. Fold change values above +1.5 represent genes upregulated in the HG-50SBM + 2SPN vs. LG-50SBM + 2SPN phenotype, while values below −1.5 represent downregulated genes in the same comparison.

### 2.7. Gene Ontology and KEGG Eenrichment Analyses

The lists of differentially expressed genes were subjected to an enrichment analysis (Biological Process, PB) with the Cytoscape ClueGO plugin (https://cytoscape.org/, on 12 October 2020) (hypergeometric test, *p*-value < 0.05) and Gene Ontology (GO) functional annotation database. Enrichment functional analysis and gene expression profiling were used to identify biological processes that were increased or decreased in the HG-50SBM + 2SPN phenotype. The analysis was carried out using the R GOplot package. The following z-score formula was used to estimate whether each enriched biological process was increased or decreased:

(1) $$z-score=\frac{Number\;of\;upregulated\;genes-Number\;of\;downregulated\;genes}{\sqrt{total\;genes}}$$

All differentially expressed genes were mapped to the Kyoto Encyclopedia of Genes of Genomes (KEGG, http://www.genome.jp/kegg, accessed on 12 October 2020) database and searched for significantly enriched KEGG pathways (hypergeometric test, *p*-value < 0.05).

### 2.8. Data Deposition

The sequencing data from this study were submitted to the Gene Expression Omnibus (GEO, https://www.ncbi.nlm.nih.gov/geo/, accessed on 1 November 2020) database under Bio Project accession number PRJNA673693.

### 2.9. Histological Analysis

Additional histological analysis was performed in 6 low-growth (94 ± 20 mg) and 6 high-growth fish (199 ± 14) fed the 50SBM + 2SPN diet and 6 fish fed the 100FM (213 ± 7 mg) diet as a negative control for enteritis. Complete fish were fixed in Bouin’s solution and subsequently subjected to histological processing. Tissues were dehydrated using a standard ethanol series to 100%, cleared in xylene, and embedded in paraffin blocks. Tissue sections were cut with microtomes to produce 3 µm-thick sections and stained with hematoxylin and eosin (H&E) and Alcian blue. Histological glass slides were prepared from each sample (10 cuts). Three pictures per glass slide were analyzed, measuring each photo threefold (18 pictures per case). The observations were performed on the middle and posterior intestines, based on the morphometric analysis reported by Ferreira et al. [43]. The following variables were measured: fold height from base to apex; fold apex width, fold base width, fold chorion area, number of goblet cells, and number of intraepithelial leukocytes (IELs) per 100 µm section. Only the mononuclear leukocytes above the basal membrane were assumed to be IELs, according to Hamidian et al. [44]. Histological photos were captured with a Leica DM2000 light microscope and Axio Vision 4.9.2.0 software, using a 20X and 40X lens. Data were checked for normality and homoscedasticity using the Shapiro–Wilk and Bartlett tests, respectively. The intestinal effects of the diets were analyzed by one-way analysis of variance (ANOVA) using the Kruskal–Wallis non-parametric test followed by Dunn’s pairwise comparisons; significant differences were found (*p*-value < 0.05).

## 3. Results

### 3.1. Growth Performance

At the population level, the fish had an average initial weight and length of 12.9 ± 6.4 mg and 9.1 ± 1.3 mm, respectively (*n* = 380 at 40dpf). At 98 dpf, significant differences were observed between treatment groups for both parameters evaluated (WG, DL) in males as well as in females. Considering only the males, WG was lower in fish fed the 50SBM + 2SPN (*n* = 201) than the 100FM diet (*n* = 203), as expected (120 ± 33 mg vs. 140 ± 31 mg, *p*-value < 0.001). The same pattern was observed for DL (11.1 ± 1.8 mm vs. 11.8 ± 1.6 mm) and SGR (4.1 ± 0.5 vs 4.5 ± 0.4), with lower values in the 50SBM + 2SPN than 100FM groups (*p*-value < 0.01). At 98 dpf, the males in all families had a consistently greater mean WG on the 100FM vs. the 50SBM + 2SNP diet (by a factor of 15–17%). These differences were not statistically significant, likely due to sample size and data variability. Importantly, the families fed the 50SBM + 2SNP vs. 100FM diets showed a genotype-by-environment interaction in response to the diets and did not maintain their initial weight rankings from 40 dpf (*p* < 0.01). This result indicates that the genetic component underlying the varying levels of tolerance to a SBM diet, favoring growth in individuals with greater tolerance.

### 3.2. Global Analysis of RNA Sequencing Data

RNA samples were obtained from five higher-growth (HG; F3-7, F3-9, F12-8; F21-1; F21-7) and six lower-growth individuals (LG; F1-8, F13-1, F13-6, F20-4, F22-3, F22-5) from these families with a genotype-by-environment interaction on 50SBM + 2SPN vs 100FM. Samples were sequenced using the Illumina platform. Sequencing data yielded a total of ~573 million paired-end reads, including ~272 million reads for the HG-50SBM + 2SPN and ~301 million reads for the LG-50SBM + 2SPN fish. RNA sequencing produced an average of ~54 million and 50 million reads per sample in the HG and LG phenotypes, respectively. After trimming and quality control, a total of ~545 million high-quality reads (Q-Phred > 30) were obtained and used to identify genes that were differentially expressed by phenotype. The total number of high-quality reads per sample ranged from ~42 million to ~58 million in HG-50SBM + 2SPN and ~43 million to ~56 million in LG-50SBM + 2SPN fish. On average, 95.13% of the high-quality reads aligned to the zebrafish reference genome GRCz11 (GenBank assembly accession GCF_000002035.6). The average total number of genes expressed in the intestine was 20,604 for all phenotypes, about 80% of all recorded genes for the species (i.e., the 25,592 annotated coding genes in zebrafish) (http://www.ensembl.org/index.html, accessed on 1 September 2020).

To evaluate the similarity of transcriptomic profiles among samples within each phenotype, PCA and correlation matrix analyses were performed. The samples were grouped, according to expression profile, with clusters of higher-and lower-growth fish. In the PCA, the HG-50SBM + 2SPN groups tended to fall in the upper left quadrant (3 of 5 samples). Most of the LG-50SBM + 2SPN groups (4 of 6 samples), along with a few samples from the HG-50SBM + 2SPN phenotype, fell in the lower left quadrant (Figure S1). Consistent with the PCA analysis, the HG-50SBM + 2SPN, and LG-50SBM + 2SPN samples had average correlation coefficients of 0.90, and 0.95, respectively, indicating a strong similarity among samples within phenotypes. Sample F1-8-LG had an average correlation coefficient of 0.25 with the other samples from the same phenotype, corroborating the divergent transcriptomic profile suggested by the PCA analysis. Therefore, sample F1-8-LG was excluded from further differential expression analysis.

### 3.3. Differential Gene Eexpression by Phenotype

The transcriptomic profiles of HG-50SBM + 2SPN and LG-50SBM + 2SPN fish were compared to identify expression patterns that may correlate with SBM tolerance. A total of 665 genes were differentially expressed by phenotype (Table S1). Of these, 298 genes were upregulated and 367 downregulated in HG-50SBM + 2SPN as compared to LG-50SBM + 2SPN fish. A volcano plot was constructed to visualize gene expression profiles associated with HG-50SBM + 2SPN fish (Figure S2).

### 3.4. Enrichment Functional Analysis for Biological Processes

The lists of differentially expressed genes in HG-vs. LG-50SBM + 2SPN fish (*n* = 665 genes) were subjected to an enrichment functional analysis (Biological Process, PB) with the Cytoscape ClueGO plugin. The magnitude of the increase or decrease in each biological process was estimated in the HG-50SBM + 2SPN fish using z-score. The enriched biological processes were visualized using the R GOplot package (*p*-value ≤ 0.05). The functional analysis identified 117 enriched biological processes, which were then categorized, according to a general gene ontology ID, giving rise to 30 enriched biological processes (*p*-value < 0.05). Of these, eleven were decreased, nine were increased, and ten were neutral in the HG-50SPN + 2SPN fish (Table S2).

**Figure 2:**
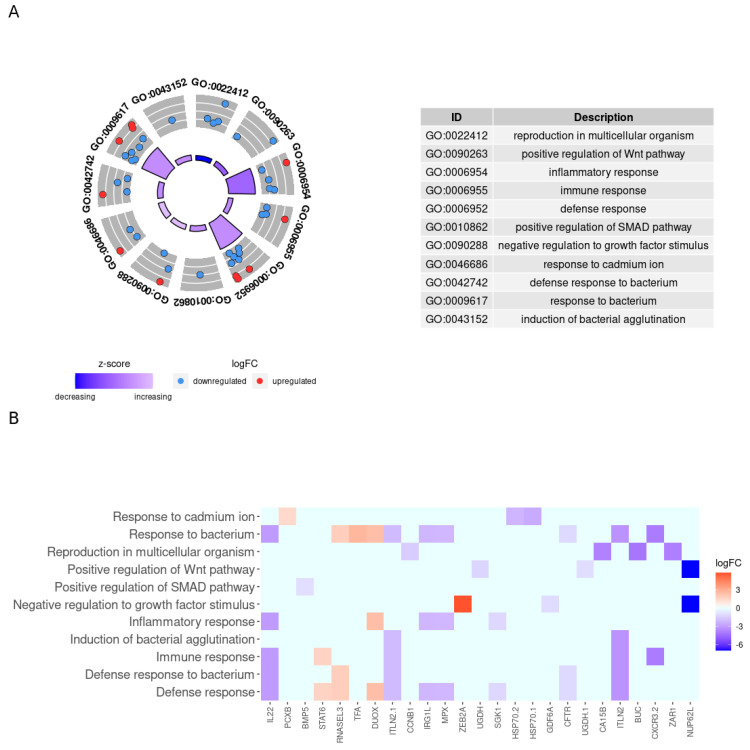
Enriched biological processes decreased in HG-50SBM + 2SPN fish and associated genes. (**A**) Circle plot of decreased biological processes in HG-50SBM + 2SPN fish. The red and blue dots on the gray ring represent the up- or downregulated genes, respectively, that participate in each process. The GO IDs of the biological processes are noted on the outer ring. The inner ring represents the z-score, and the color intensity indicates the degree to which the enriched biological process is decreased. The table on the right shows the name of each biological process by GO ID. (**B**) Heatmap of the 25 genes participating in biological processes decreased in HG-50SBM + 2SPN fish. Genes are presented as squares. Red indicates an upregulated gene, while blue indicates a downregulated gene.

Figure 2 shows the enriched biological processes that were decreased in HG-50SBM + 2SPN fish (z-score between −2.0 and −0.6) and the associated genes; a description of each process is on the right-hand side of the Figure 2A. Within this group, it is noteworthy that more than half of the biological processes were associated with immunity, and most of these genes were downregulated: GO00006954, “inflammatory response”, (z-score = −1.3) involving five genes (*irg1l*, *sgk1*, *il-22*, *mpx*, downregulated; *duox* upregulated); GO00006952, “defense response”,(z-score = −1.0) involving nine genes (*itln2.1, cftr*, *itln2*, *irg1l*, *sgk1*, *il22*, *mpx*, downregulated*; stat6*, *rnasel3,* and *duox*, upregulated); GO00006955, “immune response”, (z-score = −1.0) involving four genes (*itln2.1*, *cxcr3.2*, *itln2*, *il22*, downregulated; *stat6* upregulated); GO00009617, “response to bacterium”, (z-score = −1.0) involving ten genes (*itln2.1*, *cxcr3.2*, *cftr*, *itln2*, *irg1l*, *il22*, *mpx*, downregulated; *duox, tfa*, *rnasel13*, upregulated); GO0042742, “defense response to bacterium”, (z-score = −1.0) involving five genes (*itln21*, *cftr*, *itln2*, *il22*, downregulated; *rnasel3*, upregulated). In three biological processes, only downregulated genes were involved: GO00043152, “induction of bacterial agglutination”, (z-score = −1.0) involving one gene (*itln2.1*, downregulated); GO00010862, “positive regulation of SMAD pathway”, (z-score = −1.0) involving one gene (*bmp5*, downregulated); and GO00090263, “positive regulation of Wnt pathways”, (z-score = −1.4) involving two genes (*ugdh.1*, *nup62l*, downregulated). Other decreased processes were GO00090288, “negative regulation to growth factor stimulus”, (z-score = −0.6) involving three genes (*gdf6a*, *nup62l*, downregulated; *zeb2a*, upregulated); and GO0046686, “response to cadmium ion”, (z-score = 0.6) involving three genes (*hsp70.1*, *hsp70.2*, downregulated; and *pcxb*, upregulated) and GO0022412, “reproduction in multicellular organism”, (z-score = −2.0) involving four genes (*zar1*, *ca15b*, *ccnb1*, *buc*, downregulated). Figure 2B shows the gene expression profiles involved in decreased biological processes.

**Figure 3:**
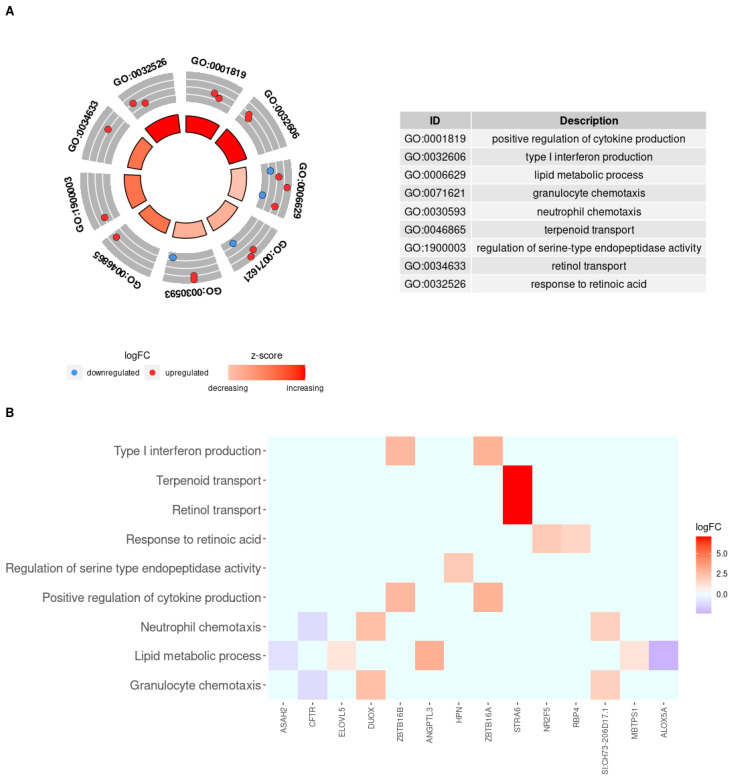
Enriched biological processes increased in HG-50SBM + SPN fish and associated genes. (**A**) Circle plot of increased biological processes in HG-50SBM + 2SPN fish. The red and blue dots on the gray ring represent the up- or downregulated genes, respectively, that participate in each process. The GO IDs of the biological processes are noted on the outer ring. The inner ring represents the z-score, where the color intensity indicates the degree to which the enriched biological process is increased. The table on the right shows the name of each biological process by GO ID. (**B**) Heatmap of the 14 genes participating in increased biological processes in HG-50SBM + 2SPN fish. Genes are presented as squares. Red indicates an upregulated gene, while blue indicates a downregulated gene.

Figure 3 shows the nine enriched biological processes that were increased in HG-50SBM + 2SPN fish (z-score value from 0.45 to 1.41) and the associated genes. Of the nine processes, three showed only a modest increase in HG fish: GO006629, “lipid metabolic process”, (z-score = 0.45) involving five genes (*mbtps1*, *angptl3*, *elovl5*, upregulated; *asah2, alox5a*, downregulated); GO0030593, “neutrophil chemotaxis”, (z-score = 0.58) involving three genes (*duox, si:ch73-206D17.1* (previous name: *styk1b*) upregulated; *cftr*, downregulated); and GO0071621, “granulocyte chemotaxis”, (z-score = 0.58) involving the same three genes. The most markedly increased processes were GO0032526, “response to retinoic acid”, (z-score = 1.41) involving two genes *(nr2f5*, *rbp4*, upregulated); GO0001819, “positive regulation of cytokine production”, (z-score = 1.41) involving two genes (*zbtb16a*, *zbtb16b*, upregulated); and GO0032606, “type 1 interferon production”, (z-score = 1.41) involving the same two genes. The moderately-increased processes were GO1900003, “regulation of serine-type endopeptidase activity”, (z-score = 1.0) involving one gene (*hpn*, upregulated); and GO0034633, “retinol transport”, (z-score = 1.0) involving one gene (*stra6*, upregulated); and GO0046865, “terpenoid transport”, (z-score = 1.0) involving the same single gene. The expression profiles and number of genes participating in the biological processes that were increased in the HG fish are presented in Figure 3B. 

Finally, ten biological processes were neutral (z-score = 0) (Figure S3). The number of up- or downregulated genes in each of these processes was equal, which results in a z-score of zero. Seven biological processes were related to intestinal immunity: GO0004686, “response to stress” (involving 20 genes); GO000965, “response to abiotic stimulus” (involving ten genes); GO0030322, “defense response to other organism” (involving six genes); GO1900003, “leukocyte migration” (involving four genes); GO0000030, “macrophage migration” (involving two genes); GO0055003, “macrophage chemotaxis” (involving two genes); and GO0003182, “reactive oxygen species metabolic process” (involving two genes). Interestingly, 16 out of 30 genes involved in these neutral processes were also involved in decreased or increased processes mentioned above.

### 3.5. KEGG Pathway Enrichment Analysis

**Figure 4:**
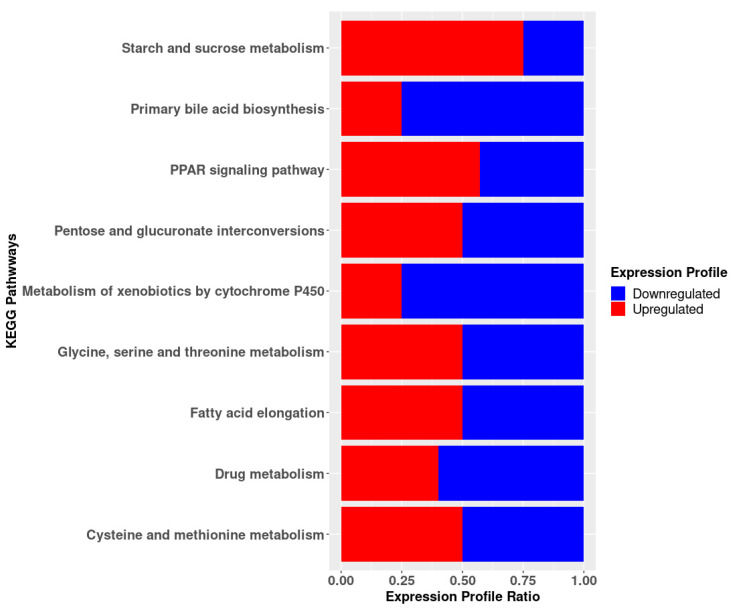
KEGG enrichment analysis from differentially expressed genes between HG-50SBM + 2SPN and LG-50SBM + 2SPN fish. Red and blue regions represent proportion of up- or downregulated genes in HG-50SBM + 2SPN, respectively.

KEGG pathway analysis was used to screen for enriched signaling pathways in HG-50SBM + 2SPN fish (Table S3). The principal pathways involving DEGs associated with lipid metabolism, including “fatty acid elongation”, “primary bile acid biosynthesis”, and “PPAR signaling pathway” (involving 12 genes: *abhd17ab*, *elovl5*, *elovl6*, *zgc:77118*, *cyp27a1.2*, *cyp27a1.4*, *cyp46a1.1*, *cyp7a1*, *acsbg1*, *lpl*, *slc27a1b*, *slc27a6)*. Other genes were associated with amino acid metabolism including “glycine metabolism, serine and threonine” and “metabolism of cysteine and methionine” (involving 7 genes: *agxt2*, *mat2ab*, *mat2al*, *si:dkey-40g16.6*, *alas1*, *dao.1*, *gldc*). Moreover, some genes were associated with metabolism of sugars, including “metabolism of starch and sucrose” and “interconversions of pentose and glucuronate” (involving 7 genes: *gck*, *treh*, *ugp2a*, *zgc:66313*, *kl*, *ugdh*, *ugt1a1*). Finally, several genes were associated with „metabolism of xenobiotics by cytochrome P450” and “metabolism of drugs” (including 5 genes: *gsta.2*, *gstt1b*, *mgst1.2*, *ugt1a1*, *ces2*) (Figure 4).

### 3.6. Intestinal Histology

**Figure 5:**
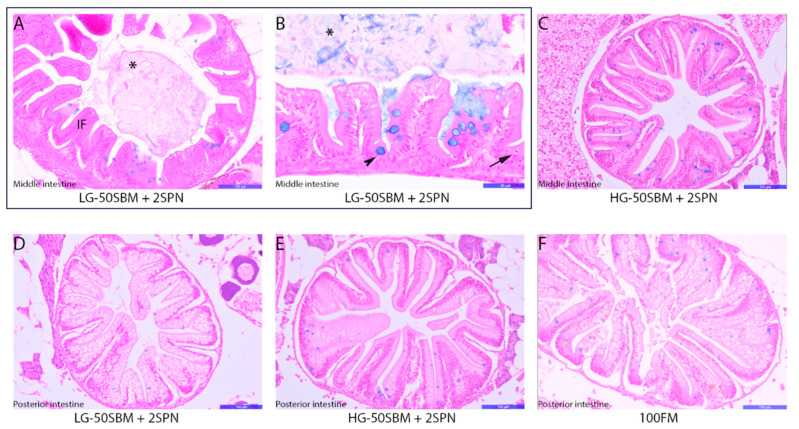
Optical microscopy of intestinal cross sections of zebrafish fed the experimental diets. Histological sections of the middle intestine of a LG-50SBM + 2SPN fish, presented inside the rectangle (**A**,**B**). Histological sections of the middle intestine of a HG-50SBM + 2SPN fish (**C**). Histological sections of the posterior intestine of LG- and HG-50SBM + 2SPN and 100FM fish (**E**,**F**). (IF) decreased intestinal fold size and enlarged intestinal lumen; (asterisk) mucus; (arrowhead) goblet cells; (arrow) intraepithelial leukocytes. The scale bar for (**A**,**C**–**F**) corresponds to 100 µm, and the scale bar for (**B**) corresponds to 50 µm.

Histology showed that the middle intestine of the LG-50SBM + 2SPN group had decreased fold height with lumen expansion as compared to the HG-50SBM + 2SPN fish (113 ± 40 vs. 151 ± 33 µm), as well as decreased fold base width (50 ± 12 vs. 64 ± 20 µm) and fold chorion area (477 ± 351 vs. 1100 ± 609 µm^2^) (*p*-value < 0.05 Table S4), reflecting harmful effects of SBM and saponins (Figure 5A,B). Values for HG-50SBM + 2SPN fish and control (100FM) fish did not differ significantly in terms of fold height, fold apex width, fold base width, or fold chorion area (*p*-value > 0.05). The HG-50SBM + 2SPN fish had a normal structural appearance (Figure 5C, Table S4). There were no significant differences between LG- and HG-50SBM + 2SPN fish for any of these variables in the posterior intestine, indicating that the pathology is localized to the middle intestine. This finding also rules out the possibility that the observed differences are due to anatomical differences between phenotypes (Figure 5D,E; Table S5). There were also no notable differences between HG-50SBM + 2SPN and control (100FM) fish for most of the posterior intestine parameters assessed, confirming a healthy intestine in the HG fish (Figure 5E,F; Table S5). The number of goblet cells did not show significant differences by treatment group for the middle (9 ± 6, 9 ± 4, 13 ± 9 in LG-50SBM + 2SPN, HG-50SBM + 2SPN, and 100FM fish, respectively) or posterior intestine (6 ± 7, 6 ± 5, 7 ± 3 in LG-50SBM + 2SPN, HG-50SBM + 2SPN, and 100FM fish, respectively). However, it should be noted that the LG-50SBM + 2SPN fish had a visible accumulation of mucin in the lumen compared to the other groups (Figure 5A,B). The LG-50SBM + 2SPN fish had a significantly lower number of intraepithelial leukocytes (IELs) in the middle and posterior intestine as compared to HG-50SBM + 2SPN fish, likely related to an immune deficiency (Figure 5B; Tables S4 and S5). There were no significant differences between HG-50SBM + 2SPN and 100FM fish for number of IELs in the middle or posterior intestine.

## 4. Discussion 

This work aimed to identify genes and biological processes that may underlie tolerance to SBM in HG-50SBM + 2SPN fish, as well as the roles of these components in intestinal immunity. To this end, the fish were selected from families that showed a genotype-by-environment interaction in response to the 50SBM + 2SNP vs. 100FM diets. Individuals with the higher (HG-50SBM + 2SPN, 170 ± 18 mg) and lower (LG-50SBM + 2SPN, 76 ± 10 mg) weight gain on the SBM-based diets, respectively, were analyzed using RNA-seq assays. Using the zebrafish as a model organism allows for standard husbandry practices and straightforward generation of multiple fish families [45]. We selected individuals from unrelated progenitors (rXY values 0–0.057) to ensure adequate genetic variability. Evidence from commercial fish indicates a direct relationship between intestinal inflammation triggered by plant-based diets and decreased growth [5,6] suggesting that genetic background may be a vital contributor to SBM tolerance. The molecular mechanisms underlying this tolerance, however, remain unclear. 

**Figure 6:**
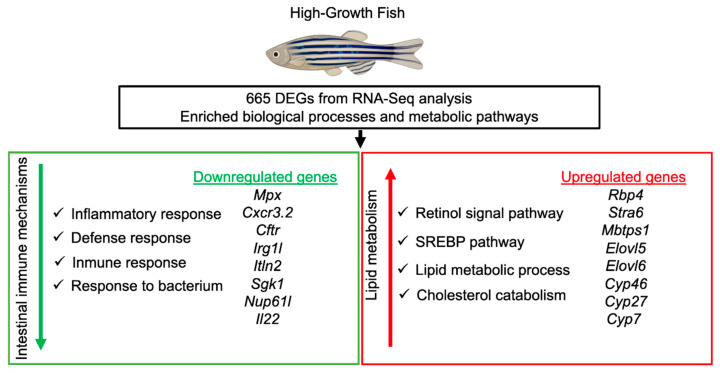
Genes and molecular mechanisms involved in intestinal tolerance to SBM-based diet in high-growth fish. Downregulation of genes associated with the inflammatory immune response and upregulation of genes related to lipid metabolism mediate SBM tolerance.

In this study, LG-50SBM + 2SPN was presumed to indicate nutritional stress linked to the low tolerance of the SBM diet. In contrast, HG-50SBM + 2SPN was assumed to indicate a greater tolerance (i.e., absence of enteritis or mild-moderate symptoms). Functional enrichment analysis identified the decreased (z score = −0.57 to −2.0) and increased (z score = 0.45 to 1.4) biological processes affected in HG-50SBM + 2SPN fish, which were linked to intestinal immunity and lipid metabolism, respectively. Figure 6 presents the enriched biological processes and metabolic pathways involved in intestinal tolerance to an SBM-based diet in HG fish. These findings suggest that these molecular mechanisms may mediate optimal growth by promoting the balance between suppressive and active pathways to maintain optimal fish health. We will discuss these biological processes in detail below. 

Early signs of inflammation include accumulation of neutrophils in the gut mucosa and polarization of macrophages and dendritic cells [46]. Natural killer cells eliminate infected cells and activate mast cells, increasing the permeability of the epithelium to amplify the inflammatory response [47,48]. In addition, differentiation of CD4+ T helper cells is critical for host defense via cytokine expression, especially Th1, Th2, Th17, Th22, and Th9 differentiation (e.g., Th17 cells express *Il-22* and *Il-17*) [49]. A SBM diet often triggers inflammation in zebrafish, generating a surge of neutrophils and macrophages in the gut and upregulation of *Il-17* and *Il-22* genes, suggesting a Th17 response [5,33]. In this study, six biological processes were markedly decreased in fish that showed optimal growth rates despite being fed the same SBM diet. All of these processes were associated with intestinal immunity: “inflammatory response”, “immune response”, “defense response”, “response to bacterium”, “defense response to bacterium”, and “induction of bacterial agglutination.” These enriched processes involved both downregulated (*mpx*, *cxcr3.2*, *cftr*, *irg1l*, *il22*, *itln2*, *itln2.1*, *sgk1*, *nup61l*) and upregulated genes (*duox*, *stat6,* and *rnasel3)*, each with a key role in regulating intestinal inflammation in fish. Specifically, the downregulated genes are related to neutrophil cell migration (*mpx*, *sgk1*) [50], leukocyte recruitment (*Irg1l*) [51], macrophage chemotaxis to the site of bacterial infection (*Cxcr3.2*) [52], mucus production (*cftr*) [53], wound healing, and protection against microbes (*il22)* [54]. The upregulated genes (*duox, stat6, rnasel3*) are involved in primary defense responses that confer larvae with the capacity to control infection [55,56]. Thus, downregulation of these genes serves primarily to suppress immune processes, indicating that HG-50SBM + 2SPN fish may not experience significant intestinal inflammation. The histological analysis reveals that in HG-50SBM + 2SPN fish, none of the parameters evaluated (fold height, fold apex width, fold base width, fold chorion area, number of goblet cells, and number of IELs) reflected any middle or posterior intestinal inflammation, with intestinal structures largely comparable to those of the 100FM group (negative inflammation control). The contrary was observed in LG-50SBM + 2SPN fish, which showed some histological effects related to inflammation, such as decreased fold height, fold base width, and fold chorion area. These results are consistent with those reported by Król et al. [7], which found that an Atlantic salmon diet containing 45% bean protein concentrate induced mild enteritis, while a 36% SBM diet generated moderate enteritis, demonstrating varying degrees of inflammation in response to the type of plant-based diet. In addition, it is apparent that some ANFs, such as saponins, can have important health consequences, causing inflammation and increasing permeability to pathogens and other unwanted substances [57]. Merrifield et al. [7] and Krogdahl et al. [58] demonstrated that a 50% SBM rainbow trout diet and 25% SBM Atlantic salmon diet increased levels of pathogenic *Psychrobacter* or *Acinetobacter* spp, respectively. Increased levels of either bacteria were associated with deformed and shortened intestinal fold morphology typical of inflammation attributable to SBM. In this study, LG-50SBM + 2SPN showed a significant decrease in number of IELs, likely related to an immune deficiency. This condition may encourage proliferation of pathogenic bacteria, associated with decreased apical width, basal width, chorion area, and intestinal length as compared to the HG-50SBM + 2SPN fish. 

On the other hand, studies in humans and snails have reported a change in gut microbiota in response to modifications of their feeding habits (e.g., omnivore-to-carnivore or herbivore-to-omnivore, respectively). Singh et al., 2017 [59] reported in humans that the intestinal microbiome plays an important role in modulating risk of intestinal inflammation or Crohn’s disease. The authors showed that a diet high in animal protein and fats and low in fiber led to a marked decrease in beneficial bacteria, such as *Bifidobacterium*, *Lactobacilli*, and *Eubacterium*, which downregulate T effector-mediated inflammation. Conversely, there were increased levels of unhealthy bacteria associated with this pathology, such as *Bacteroides* and *Enterobacteria*.

“In the snail (*Planorbella trivolvis*), an herbivorous species, switching from alfalfa to an omnivorous diet (composed of fish meal, wheat meal, yeast, germ meal, shrimp meal, and vitamin and mineral supplements) induced a drastic decrease in bacterial species, such as *Cloacibacterium*, *Pseudomonas*, and *Rhodobacter*, which are associated primarily with degradation of fibers highly present in the vegetal diet. Moreover, the omnivorous diet resulted in increased levels of the bacterial species *Aeromonas*, provoking detachment of micro-villi from simple columnar epithelium cells in the intestine [60]. Similar changes have been documented in the gut microbiota of reptiles [61] and fish [58]. Our results demonstrate that the changes in intestinal morphology were driven by the components of the SBM diet; however, analysis of the synergic effect between the intestinal microbiota and components of the SBM diet in contrasting phenotypes would be helpful to clarify this point. The most markedly increased processes in HG-50SBM + 2SPN fish, all associated with upregulated genes, were “response to retinoic acids (RA)” (*rbp4*, *nr2f5*), “retinol transport”, and “terpenoid transport” (*stra6).* Retinol (vitamin A) is essential for vision and immunity, playing a key role in regulating metabolism, differentiation, and growth [62]. Retinol circulates in the blood bound to retinol-binding protein (RBP4, encoded by *rbp4*) [63]. Retinol-bound (holo-) RBP4 is recognized by the receptor stimulated by retinoic acid 6 (STRA6, encoded by *stra6*), which transports retinol into cells [64]. Additionally, STRA6-mediated retinol transport induces JAK/STAT3/5 cascade activation [63]. This cascade, in turn, provokes expression of STAT target genes, including suppressor of cytokine signaling 3 (SOCS3), inhibiting the insulin response and leading to increased amounts of glucose available for growth [65]. In this study, *rbp4* and stra6 expression was upregulated in HG-50SBM + 2SPN fish (3-fold and 133-fold changes, respectively). This finding suggests that the receptor may contribute significantly to vitamin A uptake and cell signaling, allowing it to control important biological functions in HG fish.

Retinol and fatty acids are known activators of PPARs (peroxisome proliferator-activated receptors), which enhance lipid accumulation [66]. The PPAR signaling pathway has several immune-regulatory properties, including promotion of resolution-phase macrophages, tolerogenic dendritic cells, and Treg formation and suppression of Th17 and Th1 differentiation [67]. Consistent with this idea, our KEGG functional analysis suggested that RA may not only mediate the increase in *rbp4* and *stra6* expression but also activate the enriched PPAR signaling pathway (*acsbg1*, *lpl*, *slc27a1b*, *slc27a6*, all upregulated) in HG-50SBM + 2SPN fish. 

Other enriched KEGG pathways were “primary bile acid biosynthesis” (*cyp46a1.1*, *cyp27a1.2*, *cyp27a1.4*, *cyp7a1*) and “fatty acid elongation” (*elovl5*, *elovl6*, *abhd17*, *zgc:77118*). Furthermore, the “lipid metabolic process” (*mbtps1*, *elovl5*, *angptl3*, *asah2*, *alox5a*) was increased in HG-60SBM + 2SPN fish. Concordant with these results, prior studies have indicated that lipids play a crucial role in immune regulation by controlling the balance between lipogenesis and lipolysis [68]. One of the main pathways involved in lipid biosynthesis is driven by SREBP-1 (sterol-regulator element-binding protein 1) [69]. SREBP-1 is activated via proteolytic cleavage by MBTPS1 (membrane-bound transcription factor peptidase site 1, encoded by *mbtps1*). Then, the SREBP-1 transcription factor domain activates expression of genes encoding enzymes required for cholesterol and fatty acid metabolism (e.g., *elovl5*, *elovl6*) [70,71]. In fish, the *elovl5* gene encodes an elongase that catalyzes the rate-limiting reaction of polyunsaturated fatty acids (PUFAs) elongation. *elovl5* expression is upregulated by SREBP-1 when PUFA availability is low [72,73,74]. *elovl6* encodes another enzyme that elongates saturated fatty acids, such as palmitic and monounsaturated fatty acids [75]. The present results show that the genes encoding the protease MBTPS1 and elongases ELOV5L and ELOV6L were upregulated in HG fish, suggesting that the SRBP1 pathway contributes to immune response modulation in these fish. This idea is supported by evidence from mice, where inflammation has been associated with low intracellular anti-inflammatory monounsaturated and polyunsaturated fatty acid content (MUFAs and PUFAs). Meanwhile, SREBP1 increases levels of these compounds, decreasing the expression of pro-inflammatory genes during the resolution phase [76]. 

Along with MUFAs and PUFAs, cholesterol plays an important role in the immune response [68,77]. Cholesterol is metabolized by cytochrome P450 enzymes (such as CYP7A1, CYP27A1, and CYP46A1), generating oxysterols with inflammatory (e.g., 7α-hydroxysterols and 27-hydroxysterols) or anti-inflammatory (e.g., 24-hydroxycholesterol) properties [78,79]. CYP7A1 and CYP27A1 catalyze the formation of 7α-hydroxysterols and 27-hydroxysterols, while CYP46A1 generates 24-hydroxycholesterol to stimulate and suppress Th17 cell differentiation, respectively [80]. In HG-50SBM + 2SPN fish, *cyp27a1.2*, *cyp27a1.4*, and *cyp7a1* were downregulated, while *cyp46a1.1* was upregulated, suggesting a metabolic redirection towards 24(S)-hydroxycholesterol, leading to suppression of Th17 differentiation. These results are consistent with those reported by Abernathy et al. [81], where liver tissues from rainbow trout tolerant to a plant diet were subjected to functional analysis. These authors documented upregulated cytochrome P450A1 and T cell signaling, which suppressed interleukin-17 receptor E-like (IL17REL) expression. This receptor functionally binds *il-17* to mediate the immune response in fish [82]. Thus, upregulation of *il-17* is directly related to intestinal inflammation found in fish that cannot tolerate plant-based diets. These authors suggest that tolerant fish display different autoimmune or other detoxification responses to utilize plant proteins, avoiding the onset of enteritis [81].

On the other hand, transcriptional analyses comparing fish fed diets high in SBM and fish on FM-based diets have been performed in Atlantic salmon [9], rainbow trout [83], European seabass (*Dicentrarchus labrax*) [84], yellowtail [85], and yellow perch [26]. Hepatic transcriptomic analysis has primarily identified responses related to the metabolic and immune pathways. Some studies reported a downregulation of lipid metabolism in response to SBM diets [24,86]. Other studies have reported upregulation of lipid metabolism, identifying genes related to sterol biosynthesis (e.g., *Cyp7a1* and *hmgcr*) and lipid transport (e.g., *elovl6*) that were upregulated in response to plant proteins [9,26,83,84]. Some variation among individuals is to be expected in terms of the degree of nutritional stress caused by plant products [24]. In light of our results, the *cyp7a1* upregulation reported in the studies mentioned above, might suggest that major metabolic compounds (e.g., CYP7A1) are redirected towards generating oxysterols with inflammatory properties (e.g., 7α-hydroxysterols) in less-tolerant fish. As SBM-based diets also may induce a decreased body pool of cholesterol or bile acids, the dual actions of CYP7A1 in the cycle (cholesterol catabolism and bile acid synthesis) must be considered in interpretating the results [9]. Thus, genes involved in the sterol biosynthesis, especially *cyp7a1*, may be a key candidate gene to evaluate in future studies on degree of intestinal tolerance in response to SBM- or other plant protein-based aquafeed.

Finally, another significantly enriched KEGG pathway to highlight is “glycine, serine and threonine metabolism” (*agxt2*, *alas1*, *dao.1*, *gldc*). The *dao.1* gene encodes for intestinal D-amino acid oxidase (DAO), which modifies the composition of microbiota and could regulate neutrophil chemotaxis in innate and adaptative immunity [87]. *D*ao.1 was downregulated in HG-50SBM + 2SPN fish, consistent with the diminished inflammatory processes noted in this phenotype. In contrast, *alas1* was upregulated. This gene encodes for 5-aminolevulinic acid synthase, the first enzyme in the pathway that controls cellular heme biosynthesis and adipogenesis [88]. The increased expression of this gene in HG-50SBM + 2SPN fish reflects enhanced mitochondrial activity [89]. In a recent study [90], expression of an isoform of this gene, alas2, was higher in larger grass carp than in smaller fish, suggesting that metabolism and oxygen demands were increased to generate the energy required in larger carp, as in our HG fish.

## 5. Conclusions

The intestinal transcriptomes of zebrafish with higher (HG) and lower growth (LG) in response to an SBM-based diet were evaluated using RNA-seq. GO and KEGG enrichment analyses revealed differential expression of genes related to biological processes, such as suppression of inflammatory immune responses. The increased intestinal tolerance demonstrated by the HG-50SBM + 2SPN group may be mediated by a redirection of lipid metabolism, since the genes upregulated in these fish are involved in retinoic acid, SREBP, and cholesterol catabolism pathways. Elevated expression of these genes may dampen the inflammatory effects associated with an SBM diet, favoring fish growth. Given that these mechanisms are conserved among fish species, the differentially expressed genes reported in this study could become useful biomarkers for comparative genomics studies and SNP identification in farmed fish. These SNPs could be used to improve early selection of fish potentially tolerant to a diet formulated with SBM or other plant ingredients that tend to alter growth and feed utilization, thus contributing to a more efficient and sustainable aquaculture production. 

## Figures and Tables

**Table 1:** Ingredients and nutrient composition of control and experimental diets.

Ingredients, G/Kg	Control Diet	Experimental Diet
	50SBM + 2SPN	100FM
Fishmeal	610	250
Soybean Meal	0	500
Wheat Meal	255	113
Starch	45	45
Fish Oil	30	60
Vitamins ^1^	15	15
Minerals ^1^	15	15
Cellulose	30	0
Soy Saponin	0	2
Total	1000	1000
Analytical Composition * (Dry Basis)		
Dry Material (%)	95.3	93.5
Digestible Protein (%)	46.4	43.5
Digestible Lipids (%)	7.8	8.4
Fiber (%)	2.5	1.1
Ash (%)	12.6	9.7
Non-Nitrogenous Extract (%)	30.5	37.1
Gross Energy (MJ/Kg) **	20.0	20.3

^1^ Recommended by National Researcher Council (NRC, 1993). * Results expressed on dry basis * (%N x 6.25). ** Measurement with calorimetric pump, isoperiodic mode, at 25 °C on dry basis.

**Table 2:** Individuals selected for RNA-seq according to weight gain at 98 dpf on the 50SBM + 2SPN diet.

Family	Familial Mean Weight Gain (Mg)	Individuals Selected For RNA-Seq	Weight Gain (Mg)	Mean Weight Gain of Individuals Selected (Mg)	Phenotype
F3 (*n* = 12)	124.2 ± 32	F3-7	162.3	171.5 ± 18.1	Higher growth (HG)
		F3-9	183.0		
F12 (*n* = 10)	137.6 ± 31	F12-8	196.5		
F21 (*n* = 7)	125.7 ± 35	F21-1	150.6		
		F21-7	165.5		
F1 (*n* = 9)	105.8 ± 33	F1-8	79.4	76.4 ± 10.3	Lower growth (LG)
F20 (*n* = 7)	106.8 ± 48	F20-4	62.6		
F22 (*n* = 6)	108 ± 30	F22-3	87.3		
		F22-5	62.7		
F13 (*n* = 8)	117 ± 28	F13-1	77.9		
		F13-6	88.6		

## Data Availability

The sequencing data from this study were submitted to the Gene Expression Omnibus (GEO, https://www.ncbi.nlm.nih.gov/geo/, accessed on 1 November 2020) database under Bio Project accession number PRJNA673693.

## Supplementary Materials

The following are available online at https://www.mdpi.com/article/10.3390/genes12050700/s1, Figure S1: Principal component analysis (PCA). The location of each sample within correlation circles reflects the similarity of the transcriptomic profiles. The plot shows individuals from higher- (HG-50SBM + 2SPN, green) and lower- (LG-50SBM + 2SPN, brown) phenotypes. The different phenotypes tended to land in different quadrants (upper and lower left). The similarity of the transcriptomic profiles within the two phenotypes was confirmed in all cases, except for sample F1-8-LG. This sample is distant from the LG average; therefore, the sample was excluded from further differential expression analysis. Figure S2: Differentially expressed genes in HG-50SBM + 2SPN versus LG-50SBM + 2SPN fish (*n* = 665). Dots represent genes with an adjusted *p*-value < 0.05 and fold change > | ± 1.5|. Red dots on the right-hand side represent genes that are upregulated in HG-50SBM + 2SPN fish (*n* = 298). Blue dots on the left-hand side represent genes that are downregulated in HG-50SBM + 2SPN fish (*n* = 367). Figure S3: Heatmap of the 30 genes participating in neutral biological processes in HG-50SBM + 2SPN fish. The expression profile of each gene is represented by a blue or red square that reflects the log fold change. The blue squares represent downregulated genes and the red squares upregulated genes participating in each biological process. The z-score results suggest that the gene expression profile is consistent with neutral activity for these biological processes (z-score = 0). Table S1: Lists of differentially expressed genes between phenotypes (*n* = 665 genes). Of these, 298 were upregulated and 367 downregulated in HG-50SBM + 2SPN as compared to LG-50SBM + 2SPN fish. Table S2: Thirty enriched biological processes categorized according to a general gene ontology. Eleven processes were decreased, nine increased, and ten neutral in HG-50SBM + 2SPN fish. Table S3: KEGG pathway analysis. Table S4: Histological variables measured in the middle intestines of LG-vs.HG-50SBM + 2SPN and 100FM fish. Table S5: Histological variables measured in the posterior intestines of LG-vs. HG-50 SBM + 2SPN and 100FM fish.
