# Supplementary material for: Intestinal Transcriptome Analysis Reveals Enrichment of Genes Associated with Immune and Lipid Mechanisms, Favoring Soybean Meal Tolerance in High-Growth Zebrafish (Danio Rerio)
Source: Genes (Basel). 2021 May 8;12(5):700. doi: 10.3390/genes12050700 (PMC8151431; doi:10.3390/genes12050700)
Supplement: Supplementary file 1 [file genes-12-00700-s001.zip › genes-1203973-SI/genes-1203973-suppl-1/Table S4 and S5_histological analysis.pdf]

genes

**Table 4.** Histological variables measured in the middle intestines of LG-vs.HG-50SBM+2SPN and 100FM fish.

*p-value <0.05; **p-value<0.01; *** p-value<0.001; **** p-value <0.0001

| Treatments Groups | Histological variables measured in middle intestine | | | | | |
|---|---|---|---|---|---|---|
| | Fold height | Fold base width | Fold apex width | Fold chorion area | Number of Goblet cells | Number of IELs |
| LG-50SBM+2SPN | 113±40 | 50±12 | 44±9 | 477±351, | 8±6 | 1±1 |
| HG-50SBM+2SPN | 151±33 | 64±20 | 48±10 | 1100±609 | 9±4 | 4±3 |
| 100FM | 152±46 | 60±15 | 55±15 | 949±614 | 9±7 | 4±2 |
| Significance | | | | | | |
| LG-vs.-HG-50SBM+2SPN | 0.0037** | 0.0419* | 0.9999 | 0.0006*** | 0.9999 | 0.0001**** |
| LG-50SBM+2SPN vs. 100 FM | 0.0351* | 0.1023 | 0.0356* | 0.0090** | 0.2051 | 0.0001**** |
| HG-50SBM+2SPN vs. 100FM | 0.9999 | 0.9999 | 0.3290 | 0.9999 | 0.7209 | 0.9999 |

**Table S5.** Histological variables measured in the posterior intestines of LG-vs. HG-50SBM+2SPN and 100FM fish.

| Treatments Groups | Histological variables measured in posterior intestine | | | | | |
|---|---|---|---|---|---|---|
| | Fold height | Fold base width | Fold apex width | Fold chorion area | Number of Goblet cells | Number of IELs |
| LG -50SBM+2SPN | 115±19 | 57±18 | 50±14 | 470±223 | 6±7 | 1±2 |
| HG -50SBM+2SPN | 122±23 | 59±21 | 48±18 | 679±395 | 6±5 | 3±2 |
| 100FM | 154±30 | 69±18 | 55±17 | 896±549 | 9±4 | 5±4 |
| Significance | | | | | | |
| LG-vs.-HG-50SBM+2SPN | 0.9999 | 0.9999 | 0.9999 | 0.2885 | 0.9999 | 0.0385 |
| LG-50SBM+2SPN vs. 100 FM | 0.0006*** | 0.1180 | 0.9999 | 0.0085** | 0.1783 | 0.0005*** |
| HG-50SBM+2SPN vs. 100FM | 0.0059** | 0.2227 | 0.3017 | 0.2610 | 0.3199 | 0.6198 |

**p-value<0.01; *** p-value<0.001
