# Supplementary figures and images for: Intestinal Transcriptome Analysis Reveals Enrichment of Genes Associated with Immune and Lipid Mechanisms, Favoring Soybean Meal Tolerance in High-Growth Zebrafish (Danio Rerio)
Source: Genes (Basel). 2021 May 8;12(5):700. doi: 10.3390/genes12050700 (PMC8151431; doi:10.3390/genes12050700)

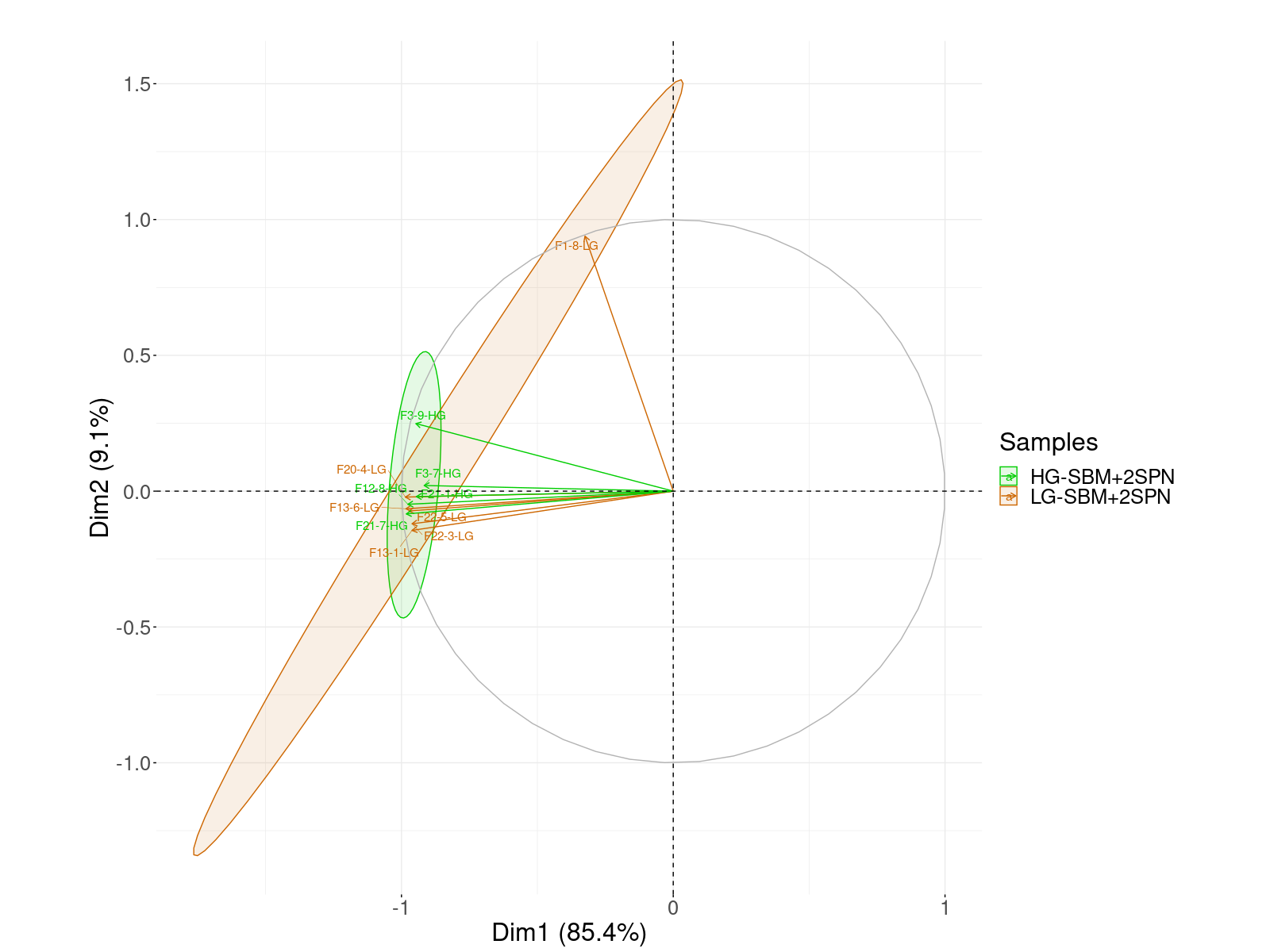

Supplement: Supplementary file 1 [file genes-12-00700-s001.zip › genes-1203973-SI/genes-1203973-suppl-1/Figure S1_PCA analysis_Elipe_T_repel_T_x1600x1200.tiff]

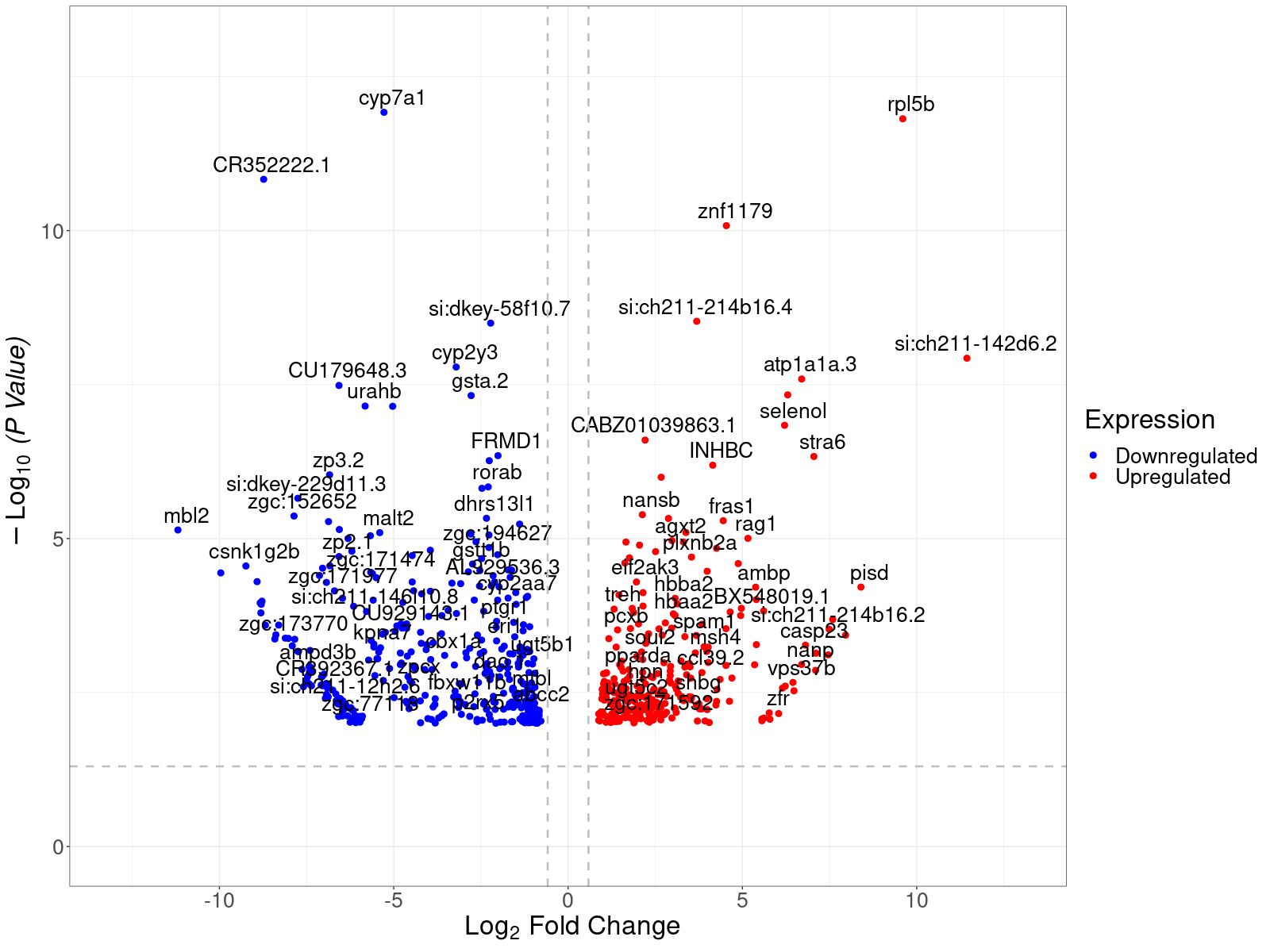

Supplement: Supplementary file 1 [file genes-12-00700-s001.zip › genes-1203973-SI/genes-1203973-suppl-1/Figure S2_Volcano Plot_1600x1200_3-2-21.tiff]

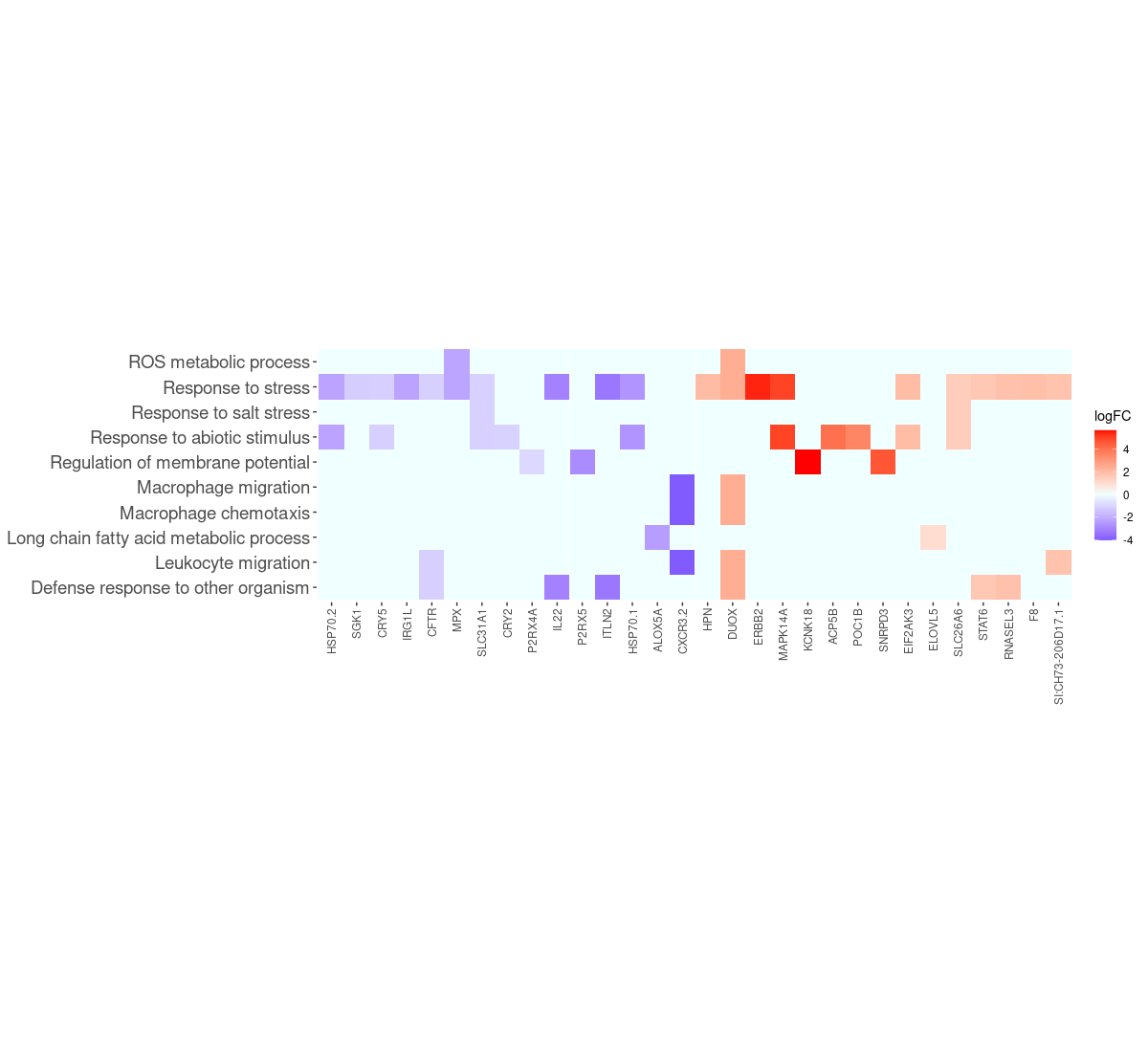

Supplement: Supplementary file 1 [file genes-12-00700-s001.zip › genes-1203973-SI/genes-1203973-suppl-1/Figure S3_Heatmap_Neutros_1200x1100.tiff]
